# Molecular Oxygen Activation by Citric Acid Boosted Pyrite–Photo–Fenton Process for Degradation of PPCPs in Water

**DOI:** 10.3390/molecules28020607

**Published:** 2023-01-06

**Authors:** Juntao Guo, Yihui Zhang, Jinjun Li, Feng Wu, Liting Luo

**Affiliations:** 1 Hubei Key Lab of Biomass Resource Chemistry and Environmental Biotechnology, School of Resources and Environmental Science, Wuhan University, Wuhan 430079, China; 2 State Key Laboratory of Magnetic Resonance and Atomic and Molecular Physics, National Center for Magnetic Resonance in Wuhan, Wuhan Institute of Physics and Mathematics, Innovation Academy for Precision Measurement Science and Technology, Chinese Academy of Sciences, Wuhan 430071, China

**Keywords:** pyrite, citric acid, sunlight, photo-Fenton, PPCPs, QSAR

## Abstract

Pyrite has been used in photo-Fenton reactions for the degradation of pollutants, but the application of photo-Fenton processes with extra H_2_O_2_ in real water/wastewater treatment has still been limited by the economic cost of H_2_O_2_ and artificial light sources. Herein, citric acid (CA) and simulated/natural sunlight are used to develop a pyrite-based photo-Fenton system (pyrite–CA–light) in situ generating H_2_O_2_ through the enhanced activation of molecular oxygen. The degradation of pharmaceuticals and personal care products (PPCPs), especially acetaminophen (APAP) as the main target pollutant, in the pyrite–CA–light system was investigated. The effects of influencing factors such as various organic acids, APAP concentration, pH, pyrite dosage, CA concentration and co-existing anions (HCO_3_^−^, Cl^−^, NO_3_^−^, SO_4_^2−^ and H_2_PO_4_^−^) were examined. At a pyrite dosage of 0.1 g L^−1^, CA concentration of 0.6 mM and an initial pH of 6.0, the degradation efficiency of APAP (30 μM) was 99.1% within 30 min under the irradiation of xenon lamp (70 W, λ ≥ 350 nm). Almost the same high efficiency of APAP degradation (93.9%) in the system was achieved under natural sunlight irradiation (ca. 650 W m^−2^). The scavenging experiments revealed that the dominant active species for degrading APAP was hydroxyl radical (HO^•^). Moreover, a quantitative structural–activity relationship (QSAR) model for pseudo-first-order rate constants (*k*_obs_) was established with a high significance (R^2^ = 0.932, *p* = 0.001) by using three descriptors: octanol–water partition coefficient (log*K*ow), dissociation constant (pK_a_) and highest occupied molecular orbital (HOMO). This work provides an innovative strategy of the photo-Fenton process for the degradation of PPCPs using natural minerals and ordinary carboxylic acid under sunlight.

## 1. Introduction

Photo-Fenton processes have been thought to be more efficient and cheaper than traditional Fenton processes with respect to iron utilization and H_2_O_2_ depletion [1]. However, the application of photo-Fenton processes with H_2_O_2_ as the initial oxidant in real water/wastewater treatment has still been limited by the economic cost of H_2_O_2_ and artificial light sources [2]. In addition, sunlight utilization is also a huge challenge to the photo-Fenton processes, because either H_2_O_2_ [3] or most unmodified iron-based catalysts are photoactive under the irradiation of UV light [4]. To overcome the limitation of economic cost, one simple strategy is in situ H_2_O_2_ generation instead of extra H_2_O_2_ addition [5]. Furthermore, if such molecular oxygen activation with a specific iron-based photoactive system can also meet the requirement of visible light response, the photo-Fenton processes may become more applicable and reliable in the real circumstances.

Molecular oxygen activation is an extremely important strategy for water/wastewater treatment because it is cheap, simple, accessible, reliable and green (without secondary contamination) [6]. A simple and basic method for oxygen activation is the oxygen reduction reaction (ORR) with various reductants (Reds) to produce reactive oxygen species (ROS), e.g., H_2_O_2_ and O_2_^•−^/HO_2_^•^ (reactions 1–6) [7]. Low valent transition metal species, especially Fe(II) and Cu(I), often play the role of reductants in such ORRs in water at near neutral and alkaline pHs [8]. For example, the co-oxidation of Fe(II) and arsenite (As(III)) by molecular oxygen occurs through Fe(II)-mediated H_2_O_2_ generation from ORR. The subsequential Fenton reaction producing HO^•^ or Fe(IV) with high oxidative power is thought to be responsible for As(III) oxidation [9].

One-electron ORR to H_2_O_2_:

(1) $$O_{2}+\mathrm{Red}\;\to {\;\mathrm{Ox}}^{+}{+O}_{2}^{•-}$$

(2) $$O_{2}^{•-}{+\mathrm{Red}+2H}^{+}\;\to {\;\mathrm{Ox}}^{+}{+H}_{2}O_{2}$$

(3) $$O_{2}^{•-}{+H}^{+}\;\to {\;\mathrm{HO}}_{2}^{•}\;(pK_{a}\;=\;4.8)$$

(4) $${2\mathrm{HO}}_{2}^{•}\;\to {\;H}_{2}O_{2}{+O}_{2}$$

(5) $$\mathrm{Net}\;\mathrm{reaction}\;\mathrm{of}\;1–3:\;\;O_{2}{+2\mathrm{Red}+2H}^{+}\;\to {\;2\mathrm{Ox}}^{+}{+H}_{2}O_{2}$$

Two-electron ORR to H_2_O_2_:

(6) $$O_{2}{+\mathrm{Red}+2H}^{+}\;\to {\;\mathrm{Ox}}^{2+}{+H}_{2}O_{2}$$

where Ox^+^ and Ox^2+^ represent the oxidized products of Red, respectively.

Pyrite (FeS_2_), as one of the common natural minerals containing Fe(II), can also be used to activate molecular oxygen for H_2_O_2_ production [10,11]. The transformation of contaminants in the presence of pyrite in natural environmental media has been reported with long periods of reactions from the perspectives of geochemistry and environmental chemistry [12]. The oxidation efficiency of contaminants in such a system, regarded as the pyrite-Fenton process, was not high enough for water/wastewater treatment [13]; although, some progresses have been made in pyrite-based advanced oxidation processes (AOPs) [14]. Usually, exogenous oxidant (H_2_O_2_ [15], CaO_2_ [16], persulfate [16,17], etc.) is still needed to gain efficient and rapid oxidation of the target contaminants. Moreover, pyrite is not very photoactive under irradiation with visible light [18,19], although it is thought of as a photocatalytic semiconductor with bandgap (E_g_ = 0.95 eV) [20]. In most pyrite–photo-Fenton systems, exogenous H_2_O_2_ is still necessary for the rapid oxidation of organic contaminants [11,21]. Therefore, boosting the in situ production of H_2_O_2_ is essential for the development of an applicable pyrite–photo-Fenton process. It is well-known that low-molecular-weight carboxylic acids (LMWACs, e.g., pyruvic acid, oxalic acid (OA), and citric acid (CA), etc.) can form complexes with Fe(III)/Fe(II) species which undergoes photolysis under visible light to produce H_2_O_2_ in the water [22,23,24,25]. Such a strategy of using oxalate as a modifier was preliminarily applied to the photocatalytic inactivation of Escherichia coli with natural pyrite under visible light [26]. Recently, Gong et al. [27] reported the enhanced oxidation of carbamazepine with three organic acids (tartaric acid, ascorbic acid and citric acid) induced by oxidized pyrite under UV–visible light.

In this work, CA was used as a modifier to boost the pyrite–photo-Fenton process for the oxidation of 10 pharmaceutical and personal care products (PPCPs) in water under visible light and sunlight as well. CA is more easily accessible than H_2_O_2_, persulfate and peracetic acid, e.g., from lemons, which makes it cheaper and simpler to be used in water/wastewater treatment in underdeveloped regions [28]. This work aims to (i) demonstrate the viability of using CA for the oxidation of various PPCPs under sunlight, (ii) understand the structure–activity relationship of PPCP oxidation and (iii) determine the utilization efficiencies of CA and energy in pyrite–photo-Fenton processes.

## 2. Results

### 2.1. Effects of Carboxylic Acids on Photodegradation of APAP Induced by Pyrite 

**Figure 1:**
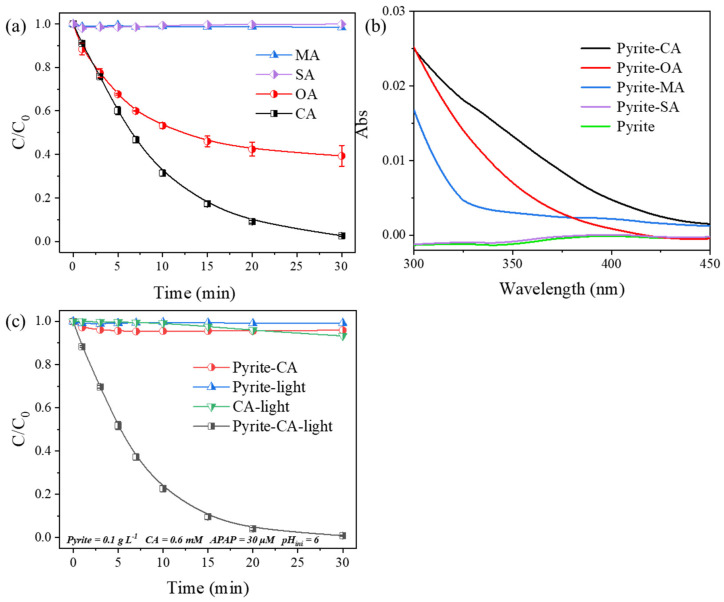
(**a**) Degradation of APAP in the presence of different low-molecular-weight carboxylic acids (maleic acid (MA), succinic acid (SA), oxalic acid (OA), and citric acid (CA)). Initial conditions: [pyrite] = 0.1 g L^−1^, [APAP] = 30 μM, [LMWCA] = 1 mM, pH_ini_ = 6. (**b**) UV–Vis absorption spectra of the reaction solutions in the presence of different low-molecular-weight carboxylic acids. Initial conditions: [pyrite] = 0.1 g L^−1^, [LMWCA] = 0.6 mM, pH_ini_ = 6. (**c**) Degradation of APAP in different systems with or without CA. Initial conditions: [pyrite] = 0.1 g L^−1^, [APAP] = 30 μM, [CA] = 0.6 mM, pH_ini_ = 6.

Four common LMWCAs (maleic acid (MA), succinic acid (SA), OA and CA) were tested to determine which is suitable for the modification of the pyrite-based photo-Fenton system. As shown in Figure 1a, the degradation efficiency of APAP is significantly enhanced by adding CA or OA, while MA and SA at the same concentration of 0.6 mM cannot improve the degradation of APAP in the presence of pyrite at pH 6. UV–Vis absorption spectra of the reaction solutions in Figure 1b show that CA and OA increase the light absorption of pyrite which facilitates the photochemical reactions in the system. Given the structure of these LMWCAs and the formation constants for their complexes with Fe(III) (Supplementary Materials Table S2), the complexing ability of these ligands and α-OH are thought to be important to the enhancement of photodegradation efficiency of APAP induced by pyrite [24,29]. As expected, CA exhibits significantly higher activity than oxalic acid in the enhancement of APAP degradation. Furthermore, CA is easily accessible and much more benign than oxalic acid to the environment; thus, it is more suitable to be used to boost the pyrite-based photo-Fenton system, namely, the pyrite–CA–light system.

Removals of APAP in the control experiments with or without CA were examined. The results in Figure 1c show that the removal efficiency of APAP with pyrite and CA is less than 5% after 30 min in the dark, indicating that the adsorption and/or oxidation of APAP is very weak. Less than 7% of APAP is removed within 30 min in the systems containing CA or pyrite alone under irradiation with simulated sunlight. Pyrite exhibits low photocatalytic activity [30,31] because of inefficient separation of the photogenerated electron–hole pairs [19]. Since CA has no absorption of simulated sunlight in the system, it alone cannot induce the photodegradation of APAP. However, in the pyrite–CA–light system, APAP degradation is significantly promoted and 99.1% APAP (initial concentration of 30 μM) is removed at 30 min. Such enhancement of APAP removal in the presence of pyrite is attributed to the combination of CA and light.

### 2.2. Effects of Main Factors

#### 2.2.1. pH

**Figure 2:**
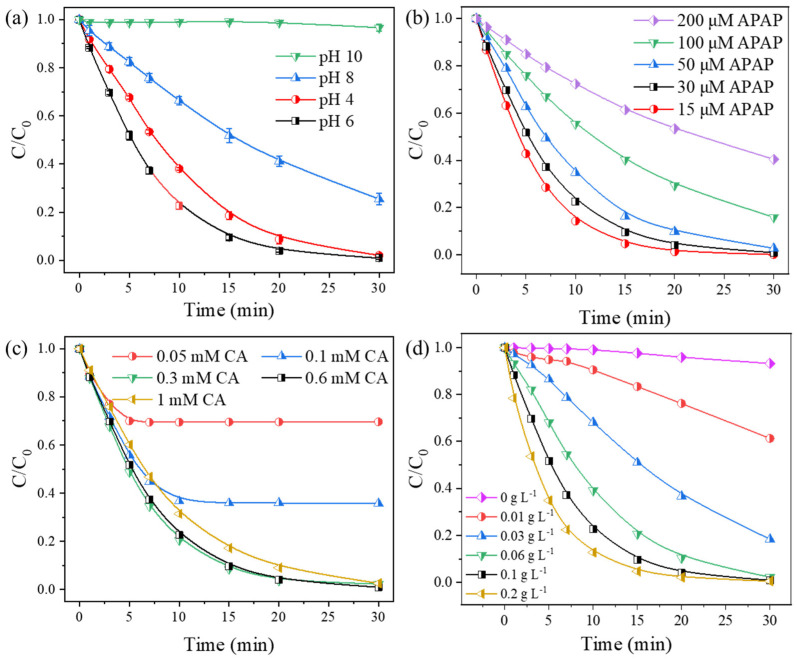
Effect of (**a**) initial pH, (**b**) initial APAP concentration, (**c**) initial CA concentration and (**d**) pyrite dosage on APAP degradation in the pyrite–CA–light system. Initial conditions: [pyrite] = 0.1 g L^−1^, [APAP] = 30 μM, [CA] = 0.6 mM, pH_ini_ = 6.

In order to investigate the effect of initial pH on APAP degradation, experiments with the pyrite–CA–light system were conducted at pH 4.0, 6.0, 8.0 and 10.0, and the changes in pH during the reactions were recorded (Supplementary Materials Figure S2). Figure 2a shows that pH 6.0 is favorable to APAP degradation, while degradation is completely suppressed at pH 10.0. For activating molecular oxygen to O_2_^•−^, there are two pathways initiated by Fe(II) (Reaction 7) and Fe(III) (Reactions 8 and 9), respectively. Near-neutral and alkaline pH facilitate Reaction 7, but a higher pH inhibits H_2_O_2_ formation through Reactions (3), (4) or (10) and the subsequent Fenton reaction (11). Meanwhile, APAP degradation at pH 4.0 is just a little slower than that at pH 6.0. Together with the fact that there is no obvious APAP degradation in the pyrite–CA system without irradiation (Figure 1c), it is concluded that Reactions (8) and (9), rather than Reaction (7), are the predominant pathways of oxygen activation in the pyrite–CA–light system. Thus, the pH dependence in Figure 2a is ascribed to the species distribution of Fe(III)/Fe(II) (Supplementary Materials Figure S3). Fe(III) mainly exists as photoactive Fe(cit)OH^−^ in aqueous solution at pH 4–8, while it exists as the much less photoactive Fe(OH)_3_(am) at pH 10.0.

(7) $$O_{2}+\mathrm{Fe}(\mathrm{II})\;\to {\;\mathrm{Fe}(\mathrm{III})+O}_{2}^{•-}$$

(8) $$\mathrm{Fe}(\mathrm{III})-\mathrm{CA}+h\nu \to {\;\mathrm{Fe}(\mathrm{II})+\mathrm{CA}}^{•-}$$

(9) $${\mathrm{CA}}^{•-}{+O}_{2}\to {\;\mathrm{CA}}_{\mathrm{ox}}{+O}_{2}^{•-}$$

(10) $$O_{2}^{•-}{+\mathrm{Fe}(\mathrm{II})+2H}^{+}\;\to {\;\mathrm{Fe}(\mathrm{III})+H}_{2}O_{2}$$

(11) $${\mathrm{Fe}(\mathrm{II})+H}_{2}O_{2}\to {\mathrm{Fe}(\mathrm{III})+\mathrm{HO}}^{•}{+\mathrm{OH}}^{-}$$

#### 2.2.2. Initial APAP Concentration

Figure 2b shows that the degradation efficiency of APAP with initial concentration of less than 50 μM is almost 100% within 30 min in the pyrite–CA–light system (0.1 g L^−1^ pyrite, 0.6 mM CA and pH 6.0), and 60.0% degradation efficiency can be achieved even for a high concentration of APAP (200 μM). The plot of the initial rate of APAP degradation (*r_0_*) versus *C*_0_ (Supplementary Materials Figure S9) presents a linear trend over the range of 15–50 μM, which follows pseudo-first kinetics, while with increasing *C*_0_ to 200 μM, *r_0_* tends to be increased in a non-linear mode. Results in Supplementary Materials Figure S4 show that the Langmuir–Hinshelwood (L–H) equation (Equation (12) [32] fits well with the data (R^2^ = 0.989), where *k_m_* is the maximum reaction rate constant (7.75 μM min^−1^) and *K* is the Langmuir constant (0.039 μM^−1^). This implies that the adsorption may play a certain role in the removal of APAP at high concentrations in the pyrite–CA–light system.

(12) $$r_{0}=\frac{k_{m}KC_{0}}{1+KC_{0}}$$

#### 2.2.3. Effect of Initial CA Concentration and Pyrite Dosage

As the source of photoactive iron species, the dosage of pyrite and CA concentration are two of the important factors in the pyrite–CA–light system. The effect of initial CA concentration was investigated over the range of 0.05–1.0 mM in the presence of 0.1 g L^−1^ pyrite at pH 6.0. Figure 2c shows incomplete removal of APAP with the initial CA being less than 0.1 mM, and degradation of APAP only occurs at the initial stages of 5 min and 10 min, respectively. By increasing the initial CA concentration to 0.3 and 0.6 mM, respectively, APAP degradation can last for 30 min and complete degradation can be achieved. By further increasing initial CA concentration to 1.0 mM, APAP degradation decreases in terms of the initial reaction rate, but the degradation efficiency at 30 min maintains above 95%. Excessive CA may compete with APAP for oxidants, thereby inhibiting APAP degradation. However, considering the treatment of a higher concentration of APAP, slightly excessive CA (i.e., 0.6 mM) was used in the following experiments.

The effect of pyrite dosage in the range of 0.01–0.2 g L^−1^ on APAP photodegradation was investigated in the presence of 0.6 mM CA at pH 6.0. As shown in Figure 2d, both degradation efficiency and the degradation rate of APAP are increased with an increasing pyrite dosage. The apparent rate constant (*k*_obs_) presents a linear increasing trend with pyrite dosage (Supplementary Materials Figure S5); no inhibition occurs with a high pyrite dosage. This indicates the good matching of the pyrite/CA ratio (0.1 g L^−1^/0.6 mM) and the applied light intensity is established in the system.

### 2.3. Mechanism of APAP Degradation in the Pyrite–CA–Light System

**Figure 3:**
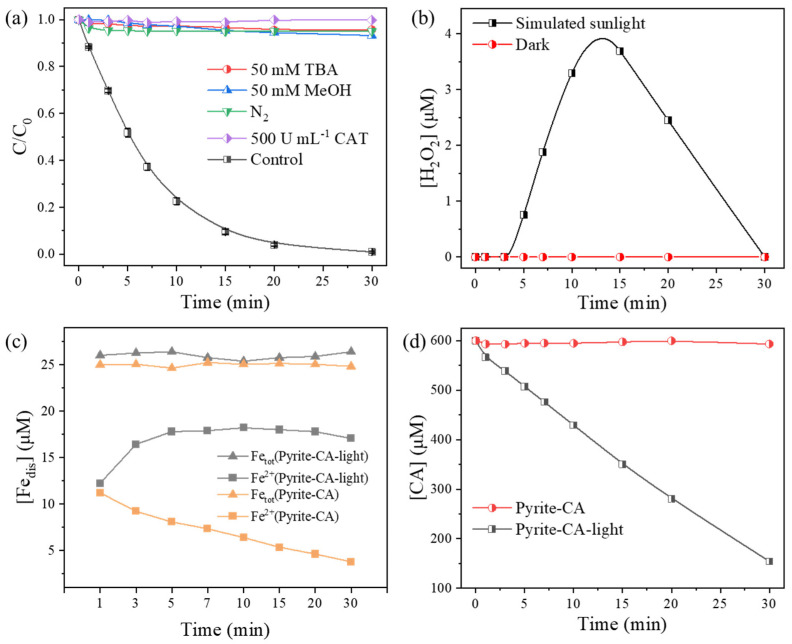
(**a**) Effect of various scavengers and anoxic solution on APAP degradation in the pyrite–CA–light system. (**b**) The concentration of H_2_O_2_ produced under simulated sunlight or in the dark. (**c**) Change in Fe(II) concentration and Fe(III) in the pyrite–CA–light system. (**d**) Change in CA concentration under simulated sunlight or in the dark. Initial conditions: [pyrite] = 0.1 g L^−1^, [APAP] = 30 μM, [CA] = 0.6 mM, pH_ini_ = 6.

As shown in Figure 3a, only 5.9% APAP removal is observed within 30 min in the atmosphere of nitrogen gas, which confirms the activation of molecular oxygen. APAP degradation is almost completely inhibited with CAT, indicating that H_2_O_2_ and/or HO^•^ might be involved. Since MeOH or TBA cannot scavenge H_2_O_2_, the complete inhibition of APAP degradation in the presence of MeOH and TBA, respectively, is attributed to their scavenges of HO^•^. Thus, the direct oxidant for APAP is HO^•^. Meanwhile, there is no difference between the inhibition resulted from MeOH and TBA, so the role of SO_4_^•−^ that is possibly produced from sulfur species in the system is excluded [33,34,35].

In order to further verify the in situ generation of H_2_O_2_, concentrations of H_2_O_2_ in the presence of pyrite and CA with or without irradiation were measured. As demonstrated in Figure 3b, the concentration of H_2_O_2_ increases first and then decreases in the pyrite–CA–light system with a maximum value of ca. 4 μM, while no H_2_O_2_ is determined in the pyrite–CA system in the dark, which is consistent with the result in Figure 1c. In addition, the terephthalic acid (TA)-trapping protocol was employed to detect the generation of HO^•^ in pyrite–CA–light. As shown in Supplementary Materials Figure S6, the fluorescence intensity increased with the process of the reaction, indicating the continuous formation of HO^•^ in the pyrite–CA–light system. To further probe the mechanism, concentrations of dissolved Fe(II) and total iron in the system with or without irradiation were determined, and the concentration of Fe(III) is the difference between the concentration of total iron and Fe(II). Interestingly, there is no significant difference between these two systems not only in the total iron (ca. 25 μM) throughout the reaction, but also in the Fe(II)/Fe(III) ratio (ca. 1:1) at the initial stage of reaction (1 min) (Figure 3c). However, the Fe(II)/Fe(III) ratio varies in different modes, i.e., Fe(II) continuously decreases in the dark, while it obviously increases first and then slightly falls back under irradiation. This difference indicates that the photoinduced Fe(II)/Fe(III) redox cycle occurs in the pyrite–CA–light system.

Furthermore, changes in CA concentration were tracked in the system in the dark and under irradiation, respectively. In the presence of pyrite, no CA degradation is observed in the dark, while 80% of CA is degraded under irradiation (Figure 3d). CA, as the sacrificial agent in the pyrite–CA–light system, can form a photoactive complex with Fe(III) and undergoes oxidative degradation (Reactions 8 and 9) initiated by a photoinduced ligand-to-metal charge transfer (LMCT). In addition, 15 uM of CA was removed for every 1 uM of APAP degraded, which implies that APAP and CA could be efficiently degraded in the pyrite–CA–light system. 

**Scheme 1:**
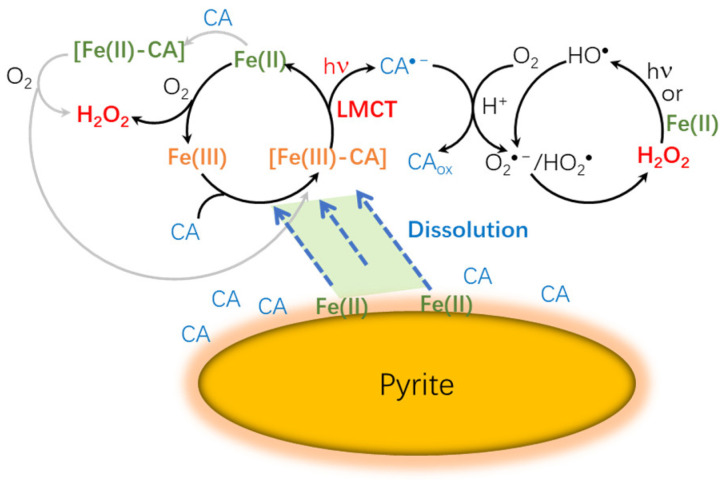
The proposed mechanism of molecular oxygen activation, in situ H_2_O_2_ generation and photo-Fenton process in the pyrite–CA–light system.

Based on the above analysis, the mechanism of molecular oxygen activation in the pyrite–CA–light system is proposed in Scheme 1. CA first combines with the Fe(III) on pyrite to form a photoactive complex (Fe(III)-CA complex), which then dissolves from pyrite into a solution. The LMCT process of Fe(III)-CA leads to the formation of Fe(II) and CA^•−^ under the irradiation of simulated sunlight, and oxygen is then activated by them to form O_2_^•−^ and H_2_O_2_. The homogeneous Fenton reaction of H_2_O_2_ with Fe(II) can generate HO^•^, and a small amount of H_2_O_2_ can also undergo photolysis under hν to generate HO^•^. Finally, APAP is degraded by a reaction with HO^•^.

### 2.4. Application of the Pyrite–CA–Light System

#### 2.4.1. Effect of Co-Existing Anions

**Figure 4:**
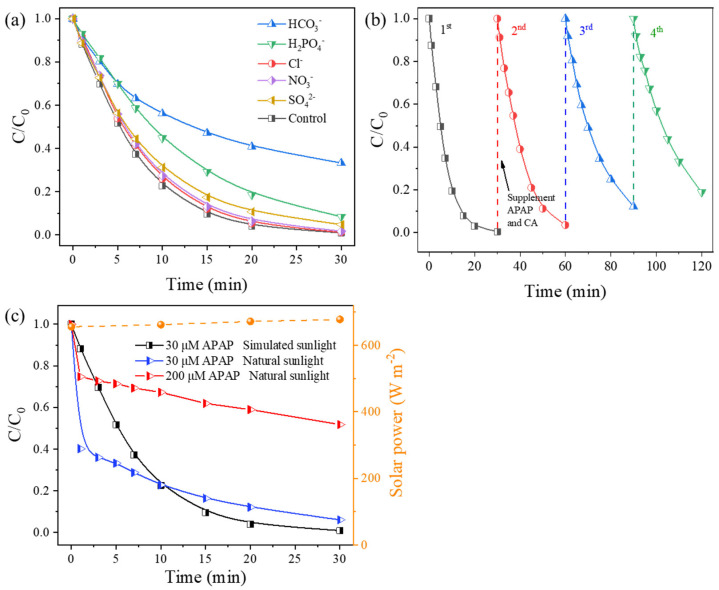
Performance of APAP degradation in the pyrite–CA–light system with (**a**) various co-existing anions, (**b**) the reused pyrite and (**c**) natural sunlight as irradiation source. Initial conditions: [Cl^−^] = [NO_3_^−^] = [SO_4_^2−^] = [H_2_PO_4_^−^] = [HCO_3_^−^] = 5 mM, [pyrite] = 0.1 g L^−1^, [APAP] = 30 μM, [CA] = 0.6 mM, pH_ini_ = 6.

In natural water and wastewaters, there are various co-existing anions which may alter the reactive species produced in the oxidation process. Figure 4a shows that Cl^−^, NO_3_^−^ and SO_4_^2−^ have no significant effect on APAP degradation or H_2_PO_4_^−^ weak inhibition, while they do on HCO_3_^−^ strong inhibition at the investigated dosage of 5 mM. Inhibition caused by H_2_PO_4_^−^ and HCO_3_^−^ is attributed to the competitive complexing against CA and scavenging HO^•^, respectively.

#### 2.4.2. Reuse of Pyrite

The reusability of pyrite is important for the application of the pyrite–CA–light system [27,36]. To simulate a practical engineering application in a sequential batch mode, APAP degradation was carried out continuously in the same reactor for four runs by adding APAP (30 μM) and CA (0.6 mM) at the end of each run of 30 min. Figure 4b shows that good performance of the system is achieved with above 95% and 80% APAP degradation in the first two runs and the second two runs, respectively. Although the degradation efficiency drops by about 10% in the third and fourth run at the reaction time of 30 min, it obviously shows an increasing trend and can be raised by prolonging the reaction time.

#### 2.4.3. Utilization of Natural Sunlight

To evaluate the effectiveness of the established system in utilizing natural sunlight, photodegradation of APAP was performed on the rooftop of the lab building under natural sunlight from 11:00 to 13:00 on 1 November 2022 (solar radiance received was about 650 W m^−2^) (Supplementary Materials Figure S8). As shown in Figure 4c, APAP degradation occurs efficiently and is much faster in the first 5 min of the reaction. At the reaction time of 30 min, degradation efficiencies of APAP at 30 and 200 μM are 93.9% and 48.2%, respectively, which are lower than those under the applied Xe lamps. Shortwave irradiation in the natural sunlight (i.e., UVB and UVA) facilitates APAP degradation in the initial stage of reactions in the pyrite–CA–light system, but it accelerates CA depletion simultaneously. This indicates that the pyrite–CA–light system is also suitable for natural sunlight conditions, but a rational strategy for maintaining a high degradation rate is replenishing CA at the proper time, especially for the purpose of treating a higher concentration of pollutants.

To evaluate the performance of the investigated system with respect to the economic cost, a simple electrical energy per order (EE/O) index was applied in this work (Text S2). Equation (13)was used to calculate EE/O, where EE/O (kWh L^−1^) is defined as the electrical energy required to remove 90% pollutant per liter, *P* is the light power (kW), *t* is the reaction time (min), *V* is the reaction volume (L), *C*_f_ and *C*_0_ are the final and the initial concentrations (μM) of APAP, respectively, [*oxidant*] is the working concentration (mM) of oxidant (the oxidant in this system is CA) and *α* is the unit convertor to translate the oxidant amount to energy unit (i.e., 2.6 × 10^−6^ kWh mg^−1^ CA). Results of estimation show that using natural sunlight can reduce EE/O by 6.29 × 10^−2^ kWh L^−1^ for APAP degradation (EE/O: 7.248 × 10^−3^ kWh L^−1^ for sunlight and 7.015 × 10^−2^ kWh L^−1^ for Xe lamps). Therefore, the pyrite–CA–light system is promising in utilizing sunlight as the energy source for the photo-Fenton process.

(13) $$\mathrm{EE}/O=\frac{\;(Pt/V)+\alpha [\mathrm{oxidant}]}{{\log(C}_{0}{/C}_{f})}\;$$

#### 2.4.4. Intermediates during the Degradation of APAP

The intermediates during the degradation of APAP are shown in Supplementary Materials Figure S6. Five intermediates, namely hydroquinone, 2-hydroxy-2-pentenedioic acid, 3-hydroxybut-3-enoic acid, 4,4-dihydroxy-3-buten-2-one and lactic acid were detected. Based on the identified intermediates, the degradation of APAP is initiated by the attack of HO^●^ on the C-N bond of its aromatic ring to the acetyl-amino group, which leads to the formation of hydroquinone. The LMCT process of the Fe(III)-CA complex leads to the degradation of CA, thus forming 2-hydroxy-2-pentenedioic acid. A subsequent attack of hydroquinone and 2-hydroxy-2-pentenedioic acid by HO^●^ forms 4,4-dihydroxy-3-buten-2-one and 3-hydroxybut-3-enoic acid, which could then be further oxidized by the reactive radicals to produce lactic acid.

Although hydroquinone is reported to cause vomiting and diarrhea in humans, it has been shown to be safe for humans at a daily ingestion level of 300–500 mg [37]. Short-chain carboxylic acids are also known to be of low toxicity or to be non-toxic [38]. The hazardous effect of 4,4-dihydroxy-3-buten-2-one is unclear. However, it is expected to be easily oxidized by HO^●^ based on this study. Thus, the degradation of APAP using the pyrite–CA–light process would be environmentally safe.

#### 2.4.5. Photodegradation of PPCPs

**Figure 5:**
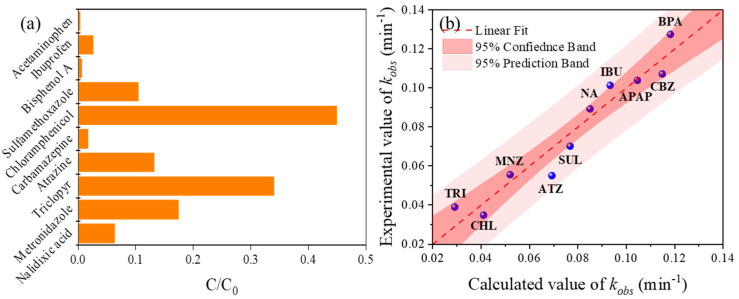
(**a**) The degradation efficiency of PPCPs in pyrite–CA–light system within 30 min. (**b**) Comparisons of calculated *k*_obs_ to experimental values for PPCPs. Initial conditions: [pyrite] = 0.1 g L^−1^, [PPCPs] = 30 μM, [CA] = 0.6 mM, pH_ini_ = 6.

Ten typical PPCPs were selected to investigate the effectiveness of the pyrite–CA–light system for treating organic pollutants. Figure 5a shows that except for carbamazepine and triclopyr, the degradation efficiency of PPCPs is above 80% within 30 min. However, the photodegradation efficiency of PPCPs varies with their molecular structures significantly. To understand the degradability of these PPCPs, the octanol–water partition coefficient (lg*K*_OW_), dissociation constant (pK_a_) and highest occupied molecular orbital (HOMO) energy were selected as descriptors for performing multiple linear regression (MLR) with *k*_obs_ to construct the quantitative structure–activity relationship (QSAR) model. lg*K*_OW_ and pK_a_ were calculated using PubChem (https://pubchem.ncbi.nlm.nih.gov (accessed on 16 December 2022)) [39], HOMO was calculated using MOPAC2016 [40] and the values of each descriptor are listed in Supplementary Materials Table S3. Finally, MLR was conducted using SPSS 22.0 software to build the QSAR model. As a result, the QSAR model (Equation (14)) is obtained where values in parentheses are 95 % confidence intervals.

(14) $$\begin{array}{c} k_{\mathrm{obs}}=0.4141\;(\pm \;0.0578)+0.0097\;(\pm \;0.0025)\;\mathrm{lg}K_{\mathrm{OW}}+0.0026\;(\pm \;0.0007)\;{\mathrm{pK}}_{a}\\ +\;0.0397\;(\pm \;0.0061)\;\mathrm{HOMO} \end{array}$$

$${n=10,\;R}^{2}=0.932,\;F=27.426,\;p=0.001$$

The relationship between the experimental and model calculated values for the ten PPCPs *k*_obs_ is shown in Figure 5b; the fitted linear over the origin tended to be the best prediction (y = x) with an R^2^ value of 0.932 and a *p*-value far below 0.05. The corresponding parameters of the *t*-tests and variance inflation factors (*VIF*) for the three molecular descriptors are listed in Supplementary Materials Table S4. This QSAR model has a superior fitting ability and indicates that the degradability of PPCPs in the pyrite–CA–light system is mainly determined by the combination of electrostatic, electron-occupied molecular orbital and hydrophobic effects of the molecules, and PPCPs with a stronger hydrophobic effect and higher-occupied molecular orbital are degraded more rapidly in the pyrite–CA–light system, which can provide instruction for the application scope of the target pollutants which can be degraded rapidly in the pyrite–CA–light system.

## 3. Materials and Methods

### 3.1. Chemicals

All chemicals were of analytical grade and were used as received. Pyrite (FeS_2_, purity > 99.9%) was purchased from Aladdin (Shanghai, China). APAP and CA were purchased from Sinopharm Chemical Reagent (Shanghai, China). The information of other chemicals is provided in the Supplementary Materials (Text S1). Ultrapure water of 18.2 MΩ cm resistivity was obtained from a water purification system (Sichuan Youpu UltraPure Technology, Chengdu, China) and was used in all experiments. All stock solutions were kept refrigerated at 4 °C and were protected from light.

### 3.2. Photochemical Procedures

The photochemical degradation of APAP and other PPCPs were carried out in a 250 mL glass-jacketed beaker with a constant temperature water circulation (25 ± 0.5 °C) [41]. The simulated light source was composed of two xenon lamps (35 W, λ ≥ 350 nm) irradiating vertically through the reactor. The irradiance spectrum is shown in Supplementary Materials Figure S1, which shows that the substantial irradiation range is mainly 400~700 nm and the UV intensity is relatively weak. In a typical experiment, 0.025 g pyrite was added into a 250 mL mixture solution containing APAP (30 μM) and CA (0.6 mM). The solution pH was adjusted to the desired value by the addition of H_2_SO_4_ or NaOH solutions. After igniting the xenon lamps, 1 mL sample was collected through a 0.22 μm PES filter at a specific time interval for subsequent analysis. An online pH meter (SIN-pH6.3, Sinomeasure, Hangzhou, China) was used to monitor the pH variations during the reaction.

### 3.3. Scavenging Experiments

The scavenging experiments were carried out to determine the predominant reactive species in the system by adding different scavengers. Methanol (MeOH), tert-butyl alcohol (TBA) and catalase (CAT) were used as scavengers for ^•^OH (plus SO_4_^•−^), HO^•^ and H_2_O_2_ [9,42], respectively. Furthermore, high-purity nitrogen gas was continuously bubbled at 1 L min^−1^ into the system to distinguish the role of molecular oxygen in the system.

### 3.4. Analytical Methods

The concentration of APAP was determined using high performance liquid chromatography (HPLC) (Agilent 1200, Agilent Technologies, Inc., Santa Clara, CA, USA). The HPLC analytical methods for APAP, other PPCPs and CA are given in Supplementary Materials Table S1. N, N-diethyl-p-phenylenediamine (DPD)-peroxidase (POD) spectrophotometry was used to quantify the H_2_O_2_ concentration. The concentration of the total iron dissolved in the supernatant was determined using an AA-7700 atomic absorption spectrometer (Shimadzu, Japan). The concentration of Fe(II) was determined via 1,10-phenanthroline spectrophotometry at 510 nm using a UV–Vis spectrophotometer (UV1601, Shimadzu, Japan). The emission spectra were analyzed with a fluorescence spectrophotometer (FS5, Edinburgh Instruments, UK), with excitation wavelength at λ = 318 nm and monitoring at λ = 426 nm, which corresponds to the main fluorescence band of the 2-hydroxyterephthalic acid (TAOH). The intermediates of APAP were detected using a gas chromatography–mass spectrometer (GC-MS) (GC-2010-GCMS-QP2010 plus, Shimadzu, Japan). The detailed information for GC-MS analysis is given in Text S3. Quality control experiments showed that the relative errors throughout this investigation were less than 10%. 

## 4. Conclusions

In summary, the pyrite–CA–light system has been established to fulfill the photo-Fenton process without extra H_2_O_2_. CA forms a photoactive complex with Fe(III) which undergoes rapid photolysis via LMCT to produce CA^•−^ and Fe(II). Both products can activate molecular oxygen to produce O_2_^•−^/HO_2_^•^ and H_2_O_2_, together with which the Fenton reagents are in situ formed in this system. Such a system can work in a wider pH range (4–8) and can treat various organic pollutants (e.g., PPCPs and CA itself as well). Target pollutants with big pK_a_ and HOMO values can be oxidized more easily. Common anions in natural waters/wastewaters, except for carbonate, have no obvious unfavorable effects on the performance of the system. Both natural sunlight and artificial light source can be used to treat organic pollutants with the established system. The utilization of sunlight makes the system greener and more cost-effective. The presented results provide a new strategy for the rational fabrication of the photo-Fenton process toward sustainable water treatment.

## Data Availability

The data that support the findings of this study are available from the authors upon reasonable request.

## Supplementary Materials

The following supporting information can be downloaded at https://www.mdpi.com/article/10.3390/molecules28020607/s1: Text S1: Materials and reagents; Text S2: Economic analysis of pyrite–CA–light system for APAP degradation; Table S1: HPLC analytical methods for used PPCPs and CA; Table S2: Comparisons between the structures of organic acids; Table S3: The relevant parameters for 10 kinds of PPCPs; Table S4: The corresponding parameters of the *t*-tests and variance inflation factors (*VIF*) for the three molecular descriptors; Figure S1: UV–Vis spectrum of xenon lamp (35 W, λ ≥ 350 nm); Figure S2: The change in pH during the reaction; Figure S3: Distributions of Fe(III) (a) and Fe(II) (b) species in the solutions containing CA at different pH values; Figure S4: Langmuir–Hinshelwood kinetic model fitted with the APAP initial concentration (15−200 μM) and *r*_0_ of the degradation process; Figure S5: Linear fitting plot fitted of the pyrite dosage (0.01−0.2 g L^−1^) versus pseudo-first-order rate constants (*k*_obs_) of the APAP degradation process.
