# Supplementary material for: Molecular Oxygen Activation by Citric Acid Boosted Pyrite–Photo–Fenton Process for Degradation of PPCPs in Water
Source: Molecules. 2023 Jan 6;28(2):607. doi: 10.3390/molecules28020607 (PMC9862748; doi:10.3390/molecules28020607)
Supplement: Supplementary file 1 [file molecules-28-00607-s001.zip › molecules-2093802-supplementary.pdf]

Supplementary Materials

Molecular Oxygen Activation by Citric Acid boosted Pyrite-Photo-Fenton Process for Degradation of PPCPs in Water

Juntao Guo [1], Yihui Zhang [1], Jinjun Li [1], Feng Wu [1,*], Liting Luo [2*]

[1] *Hubei Key Lab of Biomass Resource Chemistry and Environmental Biotechnology, School of Resources and Environmental Science, Wuhan University, Wuhan, 430079, China*

[2] *State Key Laboratory of Magnetic Resonance and Atomic and Molecular Physics, National Center for Magnetic Resonance in Wuhan, Wuhan Institute of Physics and Mathematics, Innovation Academy for Precision Measurement Science and Technology, Chinese Academy of Sciences, Wuhan 430071, China*

Content

List of texts:

Text S1. Materials and reagents.

Text S2. Economic analysis of pyrite-CA-light system for APAP degradation.

Text S3. Gas chromatography-mass spectrometer analytical method

List of tables:

Table S1. HPLC analytical methods for used PPCPs and CA.

Table S2. Comparisons between the structures of organic acids.

Table S3. The relevant parameters for 10 kinds of PPCPs.

Table S4. The corresponding parameters of the t-tests and variance inflation factors (VIF) for the three molecular descriptors.

List of figures:

Figure S1. UV-Vis spectrum of Xenon lamp (35 W, $\lambda \geq 350$ nm).

Figure S2. The change of pH during the reaction.

Figure S3. Distributions of Fe(III) (a) and Fe(II) (b) species in the solutions containing CA at different pH values.

Figure S4. Langmuir-Hinshelwood kinetic model fitted with the APAP initial concentration (15−200 μM) and r_0 of the degradation process.

Figure S5. Linear fitting plot fitted of the pyrite dosage (0.01−0.2 g L^{-1}) versus pseudo-first-order rate constants (k_{obs}) of the APAP degradation process.

Figure S6. The change of the $HO^{\bullet}$ in Pyrite-CA-light system.

Figure S7. GC-MS scan of intermediates during APAP degradation in the Pyrite-CA-light system.

Figure S8. Photograph of the setup used for the natural sunlight experiment

Figure S9. Linear fitting plot of with the APAP initial concentration (15−50 μM) and r_0 of the degradation process.

Text S1. Materials and reagents.

All chemicals were of analytical grade and were used without further purification. $Fe_2(SO_4)_3$ was purchased from Guangdong Taishan Chemical Co., Ltd. and was used as the iron source. NaOH, H_2SO_4, Na_3PO_4, $NaNO_3$, NaCl, $NaHCO_3$, Na_2SO_4, $FeSO_4$, H_2O_2 (30 wt%), lactic acid, succinic acid, oxalic acid, Na_2EDTA, MeOH, TBA, and Bisphenol A (BPA) were purchased from Sinopharm Chemical Reagent Co., Ltd. (Shanghai, China). Malic acid, H_3PO_4, *N*,*N*-diethyl-*p*-phenylenediamine (DPD), peroxidase (POD) from horseradish, 1,10-phenanthroline, Chloramphenicol (CHL), sulfamethoxazole (SUL), ampicillin (AMP), Metronidazole (MNZ), nalidixic acid (NA), Carbamazepine (CBZ) and Triclopyr (TRI) were purchased from Aladdin Industrial and Biochemical Technology Co., Ltd. (Shanghai, China). Atrazine (ATZ) and Ibuprofen (IBU) were purchased from Macklin (Shanghai, China). Catalase (CAT) was purchased from Yuanye Biotechnology (Shanghai, China).

Text S2. Economic analysis of pyrite-CA-light system for APAP degradation.

The unit conversion (α) is expressed as follows: the price of CA on Alibaba.com is \$500 per ton. Considering the average electricity cost is around 19 cent/kWh in China, one milligram of CA can be converted to 2.6316×10^{-6} kWh. As for sulfite, the price of Na_2SO_3 on Alibaba.com is \$285 per ton. Hence, one milligram of Na_2SO_3 can be converted to 1.5×10^{-6} kWh.

Under irradiation of the xenon lamp

$$\mathrm{EE/O} = \frac{\frac{2 \times 35\mathrm{W} \times 10^{-3} \times 30\mathrm{min}}{0.25\mathrm{L} \times 60\mathrm{min}} + 2.63 \times 10^{-6}\mathrm{kWh/mg} \times 0.6\mathrm{mM} \times 192.13\mathrm{g/mol}}{\lg\left(\frac{1}{0.01}\right)}$$

$\mathrm{EE/O} = 7.015 \times 10^{-2}\ \mathrm{kWh\ L^{-1}}$.

According to the Eq. (16),

$$\mathrm{EE/O} = \frac{(STt/V) + \alpha[oxidant]}{\log(C_0/C_f)} \tag{S1}$$

Where S is the bottom area of the reaction container, m^2; and T is the power of light, W m^{-2}. P can be expressed as the product of S and T, the Eq. (16) is rewritten as Eq. S1.

Under natural sunlight irradiation,

$$\mathrm{EE/O} = \frac{\frac{\pi(45\times10^{-3}\mathrm{m})^2 \times 660\mathrm{W/m^2} \times 30\mathrm{min}}{0.25\mathrm{L} \times 60\mathrm{min} \times 10^3} + 2.63 \times 10^{-6}\mathrm{kWh/mg} \times 0.6\mathrm{mM} \times 192\mathrm{g/mol}}{\lg\left(\frac{1}{0.06}\right)}$$

$\mathrm{EE/O} = 7.248 \times 10^{-3}\ \mathrm{kWh\ L^{-1}}$.

* The bottom radius of the reaction vessel used is 45 mm and the average power of light of APAP degradation experiment under natural sunlight irradiation was 660 W m^{-2}.

Text S3. Gas chromatography-mass spectrometer analytical method

The samples were thoroughly mixed with ethyl acetate, and then the organic phase inside was collected and mixed with anhydrous sodium sulfate. The dehydrated organic phase was collected and concentrated by nitrogen sweeping. The residue was derived by BSTFA/TMCS (99:1, v/v) at 60 °C for 3 h. The intermediates of APAP were identified using gas chromatography-mass spectrometer (GC-MS) (GC-2010-GCMS-QP2010 plus, Shimadzu, Japan) equipped with an Agilent DB-5MS column (30.0 m × 250 μm × 0.25 μm). The injector and detector were both kept at 250 °C. The temperature procedure of GC was as follows: the initial column temperature was 80 °C and held for 2 min, then increase to 150 °C at a rate of 7 °C/min, and held at 150 °C for 5 min. Subsequently, the temperature was increased at a rate of 7 °C/min to 250 °C and then maintained for 5 min. The injection volume is 1 μL in the splitless mode. The MS detector was operated in the EI mode (70 eV).

Table S1. HPLC analytical methods for used PPCPs and CA.

NO.	Substances	Mobile phase (v/v)	Flow rate / $mL\cdot min^{-1}$	Wavelength / nm
1	Acetaminophen	0.1% Acetic acid/Methanol = 70/30	1	243
2	Metronidazole	0.1% Acetic acid/Acetonitrile = 80/20	1	318
3	Nalidixic acid	0.1% Acetic acid /Acetonitrile = 50/50	1	257
4	Chloramphenicol	0.1%Acetic acid/Acetonitrile = 60/40	1	275
5	Sulfamethoxazole	0.1%Acetic acid/Acetonitrile = 50/50	1	272
6	Carbamazepine	0.1%Acetic acid/Acetonitrile = 50/50	1	278
7	Triclopyr	0.1%Acetic acid/Acetonitrile = 50/50	1	293
8	Atrazine	0.1%Acetic acid/Acetonitrile = 50/50	1	225
9	Bisphenol A	0.1%Acetic acid/Methanol = 35/65	1	230
10	Ibuprofen	0.1%Acetic acid/Methanol = 25/75	1	220
11	Citric acid	0.1% H_3PO_4/Methanol = 96/4	1	210

Table S2. Formation constants for 1:1 Fe(III) complexes with low-molecular-weight carboxylic acids (as HL^- or HL^{2-}). [a]

NO.	Organic acids	Structure	$\log K_{Fe(III)\text{-}LMWCA}$
1	Citric acid	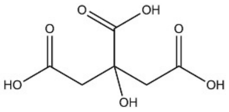	11.9
2	Oxalic acid	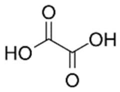	9.4
3	Maleic acid	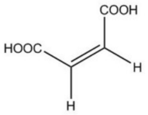	5.2
4	Succinic acid	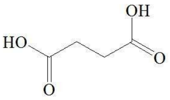	7.5

[a] Xingyun Huang, Ying Peng, Jing Xu, Feng Wu, Gilles Mailhot, Iron(III)-induced photooxidation of arsenite in the presence of carboxylic acids and phenols as model compounds of natural organic matter. Chemosphere 263 (2021) 128142

Table S3. The relevant parameters for 10 kinds of PPCPs

NO.	Chemicals	k_{obs}	lgK_{OW}	pK_a	HOMO
1	Acetaminophen (APAP)	0.1039	0.46	9.38	-8.52
2	Atrazine (ATZ)	0.055	2.34	1.6	-9.37
3	Bisphenol A (BPA)	0.1274	3.32	9.6	-8.89
4	Carbamazepine (CBZ)	0.1072	2.45	13.9	-9.04
5	Chloramphenicol (CHL)	0.0349	1.14	12.93	-10.52
6	Ibuprofen (IBU)	0.1013	3.97	5.3	-9.4
7	Metronidazole (MNZ)	0.0555	-0.02	15.42	-10.12
8	Nalidixic acid (NA)	0.0892	1.59	8.6	-9.24
9	Sulfamethoxazole (SUL)	0.0701	0.89	5.7	-9.09
10	Triclopyr (TRI)	0.039	-0.45	2.68	-9.77

Table S4. The corresponding parameters of the *t*-tests and variance inflation factors (*VIF*) for the three molecular descriptors.

Descriptor	*t*	*VIP*
$\lg K_{OW}$	3.913	1.111
pK_a	3.453	1.053
HOMO	6.498	1.155

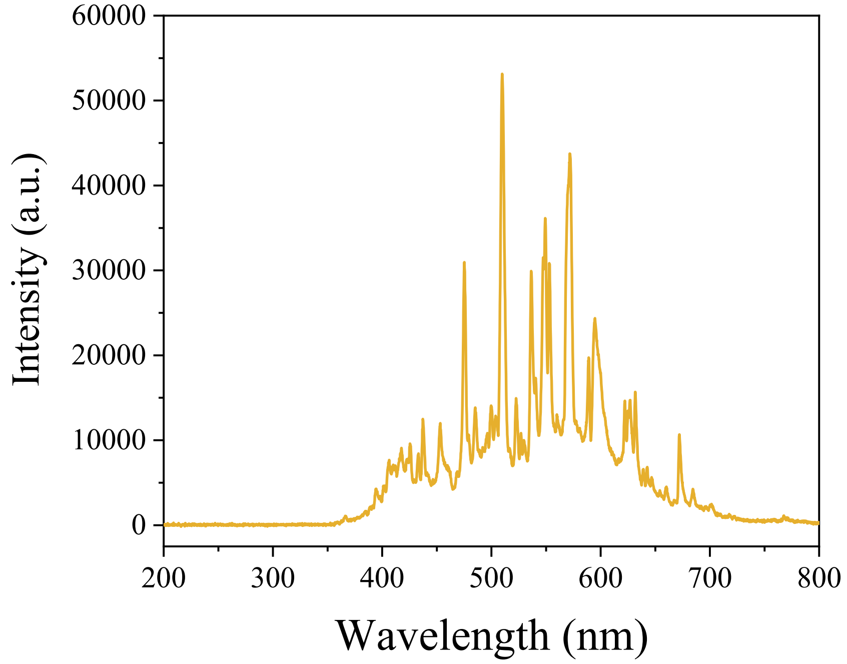

Figure S1. UV-Vis spectrum of Xenon lamp (35 W, λ ≥ 350 nm).

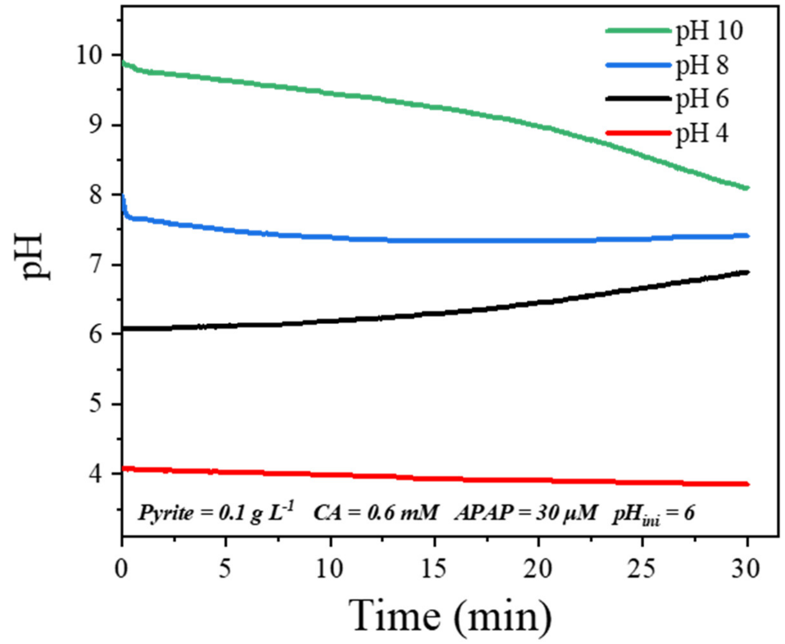

Figure S2. The change of pH during the reaction.

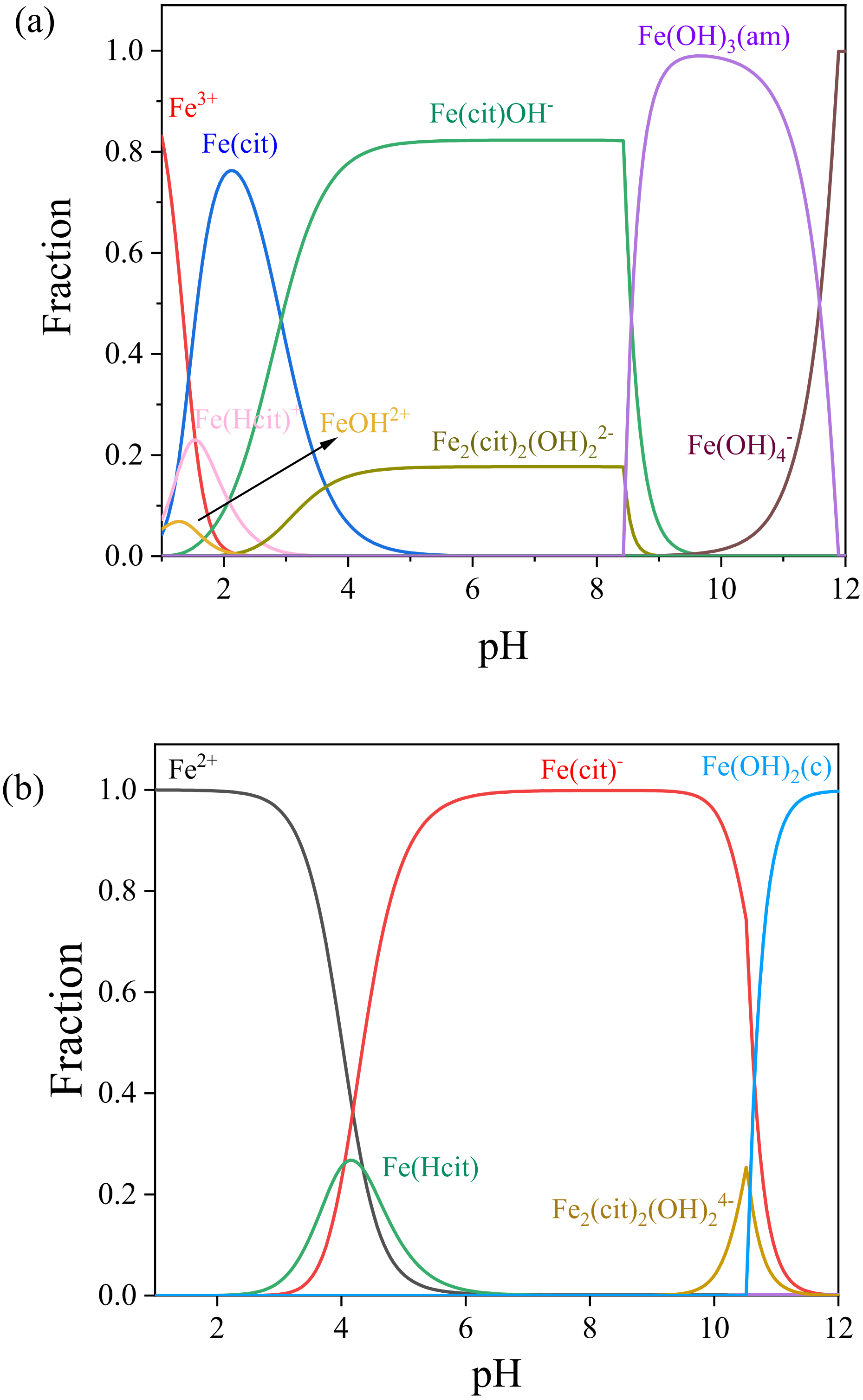

Figure S3. Distributions of Fe(III) (a) and Fe(II) (b) species in the solutions containing CA at different pH values. Initial conditions: [Fe(III)] = 15 μM, [Fe(II)] = 10 μM, [CA] = 0.6 mM.

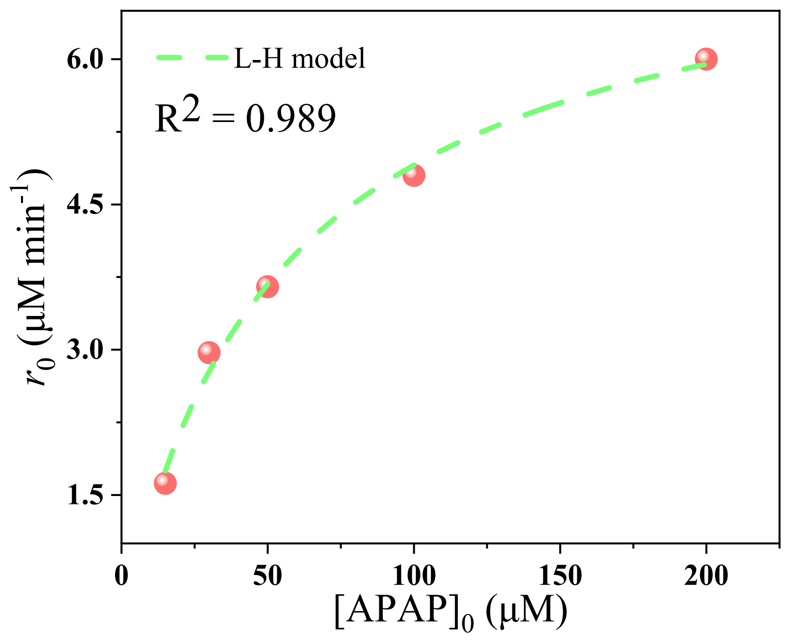

Figure S4. Langmuir-Hinshelwood kinetic model fitted with the APAP initial concentration (15−200 μM) and r_0 of the degradation process.

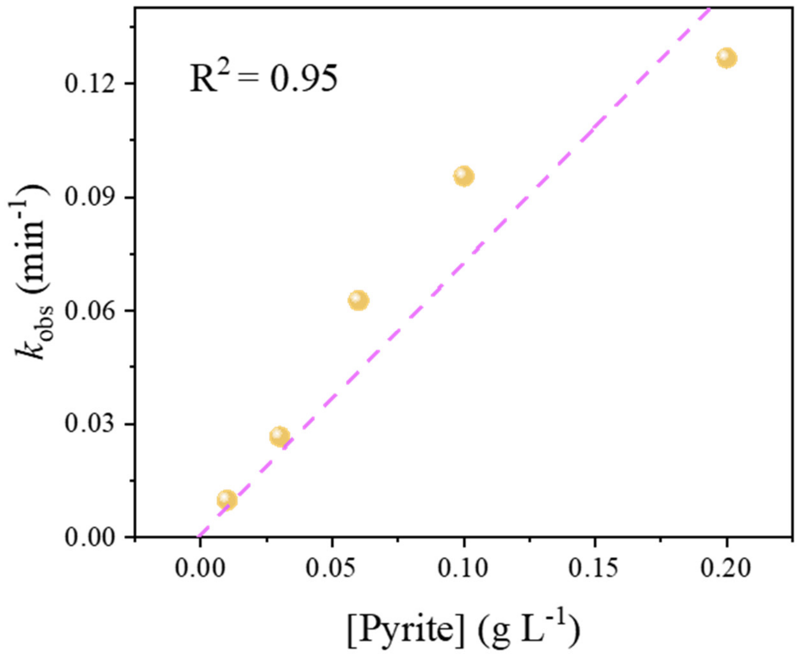

Figure S5. Linear fitting plot fitted of the pyrite dosage (0.01−0.2 g L^{-1}) versus pseudo-first-order rate constants (k_{obs}) of the APAP degradation process.

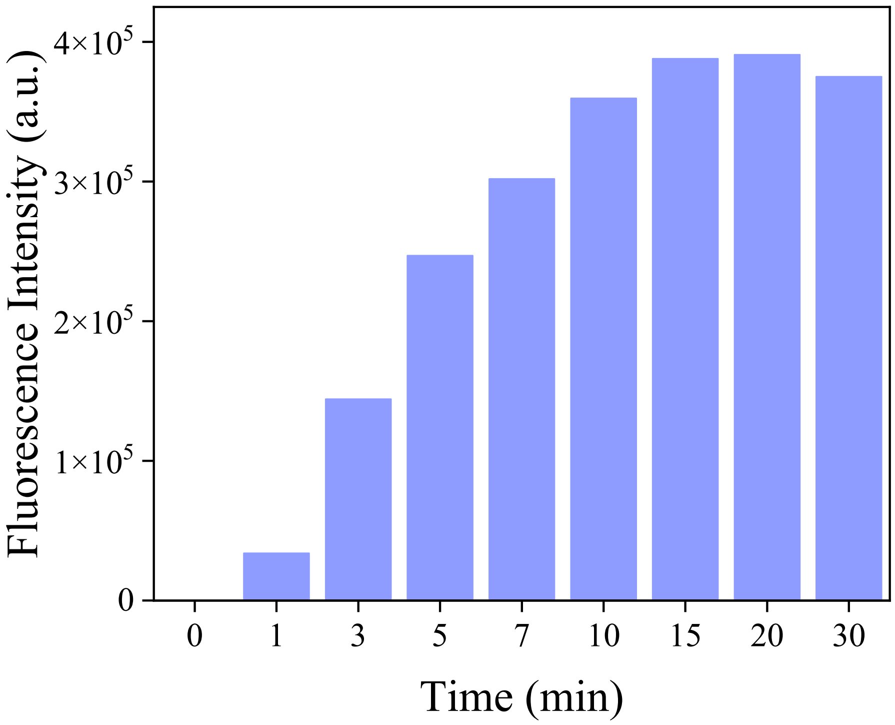

Figure S6. The change of fluorescence intensity at 426 nm with reaction time in Pyrite-CA-light system. Initial conditions: [Pyrite] = 0.1 g L^{-1}, [Terephthalic acid] = 0.5 mM, [CA] = 0.6 mM, pH_{ini} = 6.

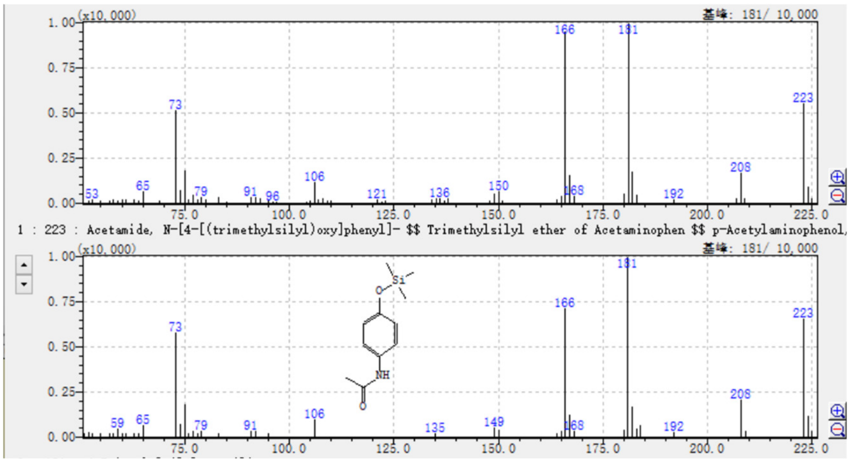
(x10,000)
基峰: 181/ 10,000
1 : 223 : Acetamide, N-[4-[(trimethylsilyl)oxy]phenyl]- $$ Trimethylsilyl ether of Acetaminophen $$ p-Acetylaminophenol,
(x10,000)
基峰: 181/ 10,000

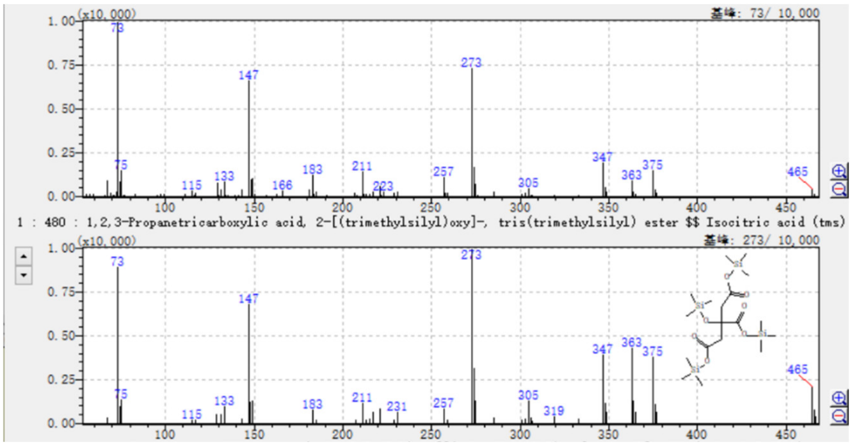
(x10,000)
基峰: 73/ 10,000
1 : 480 : 1,2,3-Propanetricarboxylic acid, 2-[(trimethylsilyl)oxy]-, tris(trimethylsilyl) ester $$ Isocitric acid (tms)
(x10,000)
基峰: 273/ 10,000

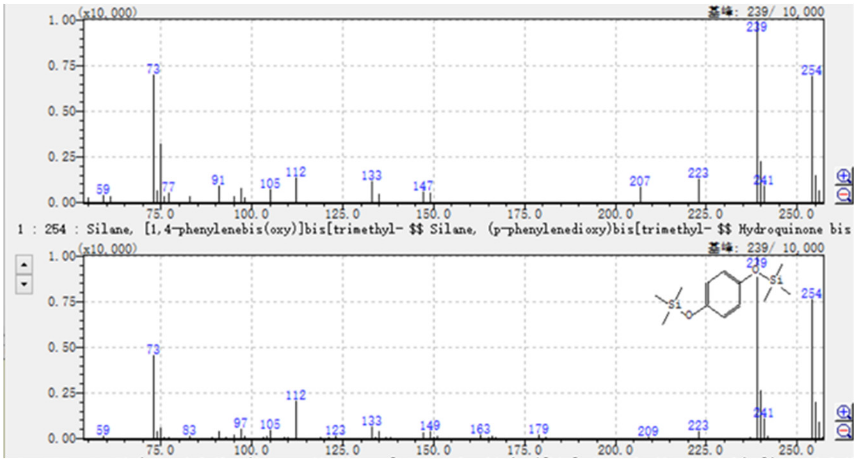
(x10,000)
基峰: 239/ 10,000
1 : 254 : Silane, [1,4-phenylenebis(oxy)]bis[trimethyl- $$ Silane, (p-phenylenedioxy)bis[trimethyl- $$ Hydroquinone bis
(x10,000)
基峰: 239/ 10,000

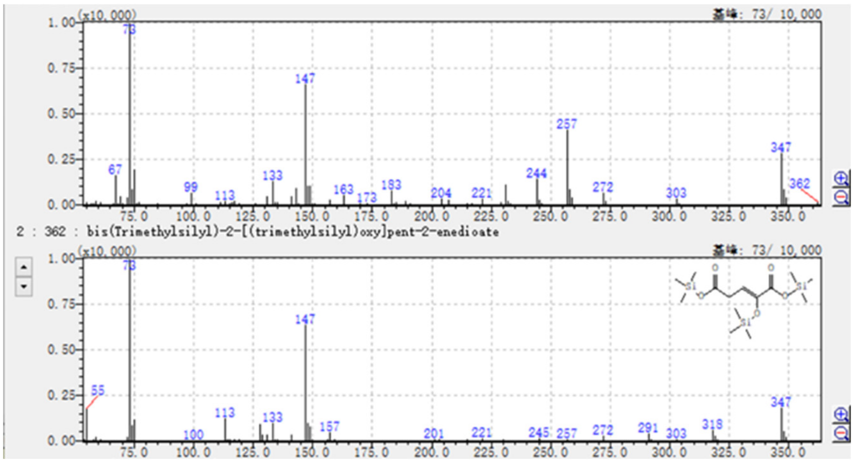
(x10,000)
基峰: 73/ 10,000
2 : 362 : bis(Trimethylsilyl)-2-[(trimethylsilyl)oxy]pent-2-enedioate
(x10,000)
基峰: 73/ 10,000

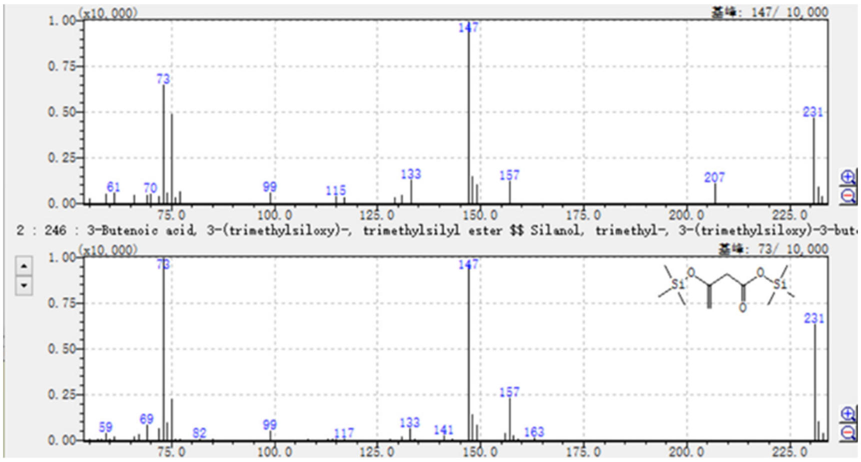
(x10,000)
基峰: 147/ 10,000
2 : 246 : 3-Butenoic acid, 3-(trimethylsiloxy)-, trimethylsilyl ester $$ Silanol, trimethyl-, 3-(trimethylsiloxy)-3-but
(x10,000)
基峰: 73/ 10,000

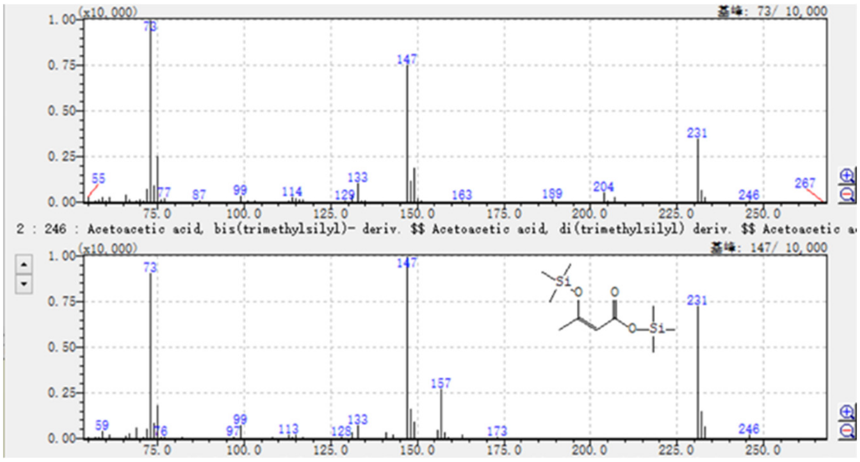
(x10,000)
基峰: 73/ 10,000
2 : 246 : Acetoacetic acid, bis(trimethylsilyl)- deriv. $$ Acetoacetic acid, di(trimethylsilyl) deriv. $$ Acetoacetic a
(x10,000)
基峰: 147/ 10,000

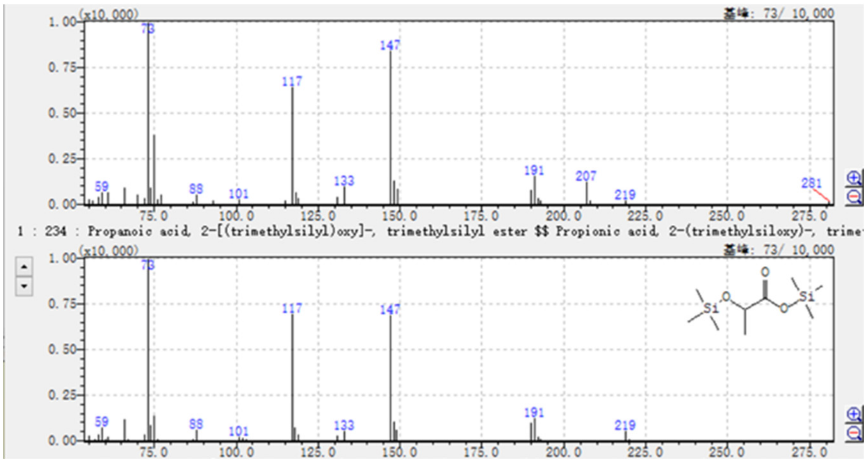

Figure S7. GC-MS scan of intermediates during APAP degradation in the Pyrite-CA-light system.

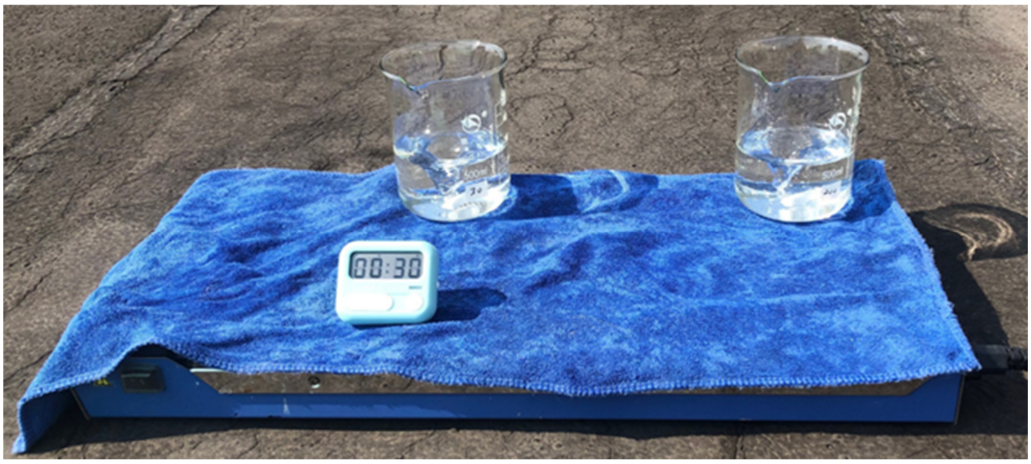

Figure S8. Photograph of the setup used for the natural sunlight experiment.

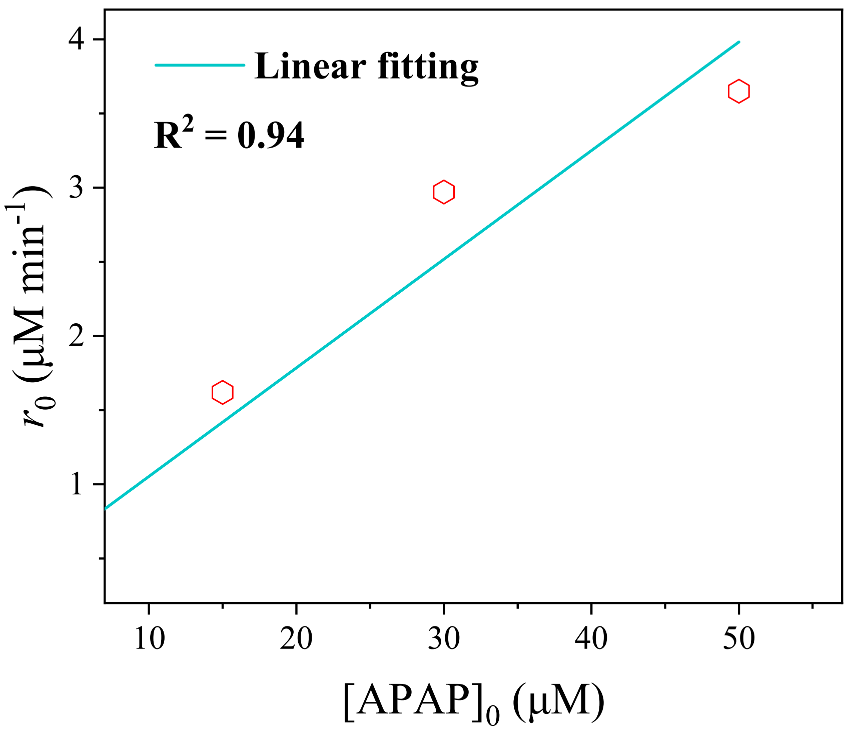

Figure S9. Linear fitting plot of with the APAP initial concentration (15−50 μM) and r_0 of the degradation process.
